# Exploratory associations between APOE-ε*4* carrier status and facial neuromuscular activity in cognitively intact adults: a cross-sectional surface electromyography study

**DOI:** 10.3389/fneur.2026.1740391

**Published:** 2026-09-23

**Authors:** Marziye Eshghi, Panying Rong, Mehrdad Dadgostar, Hyunmin Shin, Brian D. Richburg, Nelson V. Barnett, David H. Salat, Steven E. Arnold, Jordan R. Green

**Affiliations:** 1 Department of Communication Sciences & Disorders, MGH Institute of Health Professions, Charlestown, MA, United States; 2 Athinoula A. Martinos Center for Biomedical Imaging, Harvard-MIT Health Sciences and Technology School, Massachusetts General Hospital, Charlestown, MA, United States; 3 Department of Radiology, Harvard Medical School, Massachusetts General Hospital, Boston, MA, United States; 4 Department of Speech-Language-Hearing: Science and Disorders, University of Kansas, Lawrence, KS, United States; 5 Neuroimaging Research for Veterans Center, VA Boston Healthcare System, Boston, MA, United States; 6 Department of Neurology, Harvard Medical School, Massachusetts General Hospital, Boston, MA, United States; 7 Speech and Hearing Bioscience and Technology, Harvard University, Cambridge, MA, United States

**Keywords:** Alzheimer's disease, APOE-ε*4* genotype, electromyography, genetic risk factor, motor unit synchronization, neuromuscular function, speech biomarkers

## Abstract

**Introduction:**

The APOE-ε*4* allele is a genetic risk factor for late-onset Alzheimer's disease (AD). Beyond cognitive decline, APOE-ε*4* affects motor function, reducing muscle strength and coordination, potentially through mitochondrial dysfunction and oxidative stress. This study examined the influence of the APOE-ε*4* allele on neuromuscular function in oral muscles involved in speech production, using surface electromyography (EMG); and evaluated the preliminary discriminatory performance of EMG-based multivariable models in differentiating APOE-ε*4* carriers from noncarriers.

**Methods:**

Forty-two cognitively intact adults (16 APOE-ε*4* carriers, 26 noncarriers) completed speech tasks while EMG was recorded from seven craniofacial muscles. Seventy EMG features including amplitude, frequency, complexity, regularity, and functional connectivity were extracted. Statistical analyses assessed genotype effects, sex differences, and correlations with blood metabolic biomarkers, alongside group comparisons on cognitive measures to confirm preserved cognition in this sample.

**Results:**

APOE-ε*4* carriers exhibited descriptive patterns consistent with increased motor unit recruitment and synchronization, indicating altered neuromuscular control and possible compensatory adjustments. Multivariable EMG-based models showed promising internal discrimination between carriers and noncarriers, whereas standard cognitive measures did not demonstrate significant APOE-ε*4*-related differences in this cohort. EMG-based measures also showed correlation with metabolic biomarkers. Genotype and genotype-by-sex effects on the EMG-derived factors were not statistically significant. However, descriptive sex-stratified patterns suggested reduced functional connectivity in female carriers and increased functional connectivity in male carriers.

**Discussion:**

These findings suggest that speech-based neuromuscular changes may serve as exploratory candidate indicators of neuromuscular vulnerability associated with APOE-ε*4* carrier status in cognitively intact adults. While this study distinguishes APOE-ε*4* carriers from noncarriers, further work is needed to determine whether these EMG features can diagnose AD, predict progression to mild cognitive impairment (MCI) or dementia, or serve as early diagnostic or prognostic biomarkers. Additionally, their prognostic value and relationship to standard cognitive measures should be confirmed in equivalent models and in larger, independent longitudinal cohorts.

## 1 Introduction

The Apolipoprotein E4 (APOE*-*ε*4*) allele is a well-established genetic risk factor for late-onset Alzheimer's disease (AD) (1), but it is not deterministically causal. Instead, APOE-ε*4* confers a dose-dependent increase in AD risk, with heterozygous carriers facing approximately a 2–3-fold greater likelihood of developing AD relative to APOE-ε*3/*ε*3* individuals, and homozygotes facing up to a 15-fold increased risk (2–6). Nevertheless, many APOE-ε*4* heterozygotes, and even a subset of homozygotes, remain free of clinical dementia, indicating that APOE-ε*4* is characterized by substantially elevated but incomplete penetrance. Consistent with this, large-scale biomarker studies demonstrate that nearly all APOE-ε*4* homozygotes develop AD-consistent amyloid and tau pathology by mid- to late life (7), supporting the view that APOE-ε*4* homozygosity represents a distinct genetic form of AD biology, even though progression to symptomatic dementia still varies across individuals. Beyond amyloid and tau, APOE-ε4 further disrupts cerebral glucose metabolism, promotes cortical atrophy, and contributes to vascular abnormalities (8–13), while also exacerbating neuroinflammation and gliosis in both human AD brains and animal models (14–17). Clinically, carriers of APOE-ε*4* face a significantly higher risk of developing mild cognitive impairment (MCI) and AD compared to non-carriers, with the presence of this allele potentially accelerating cognitive decline and leading to earlier symptom onset (18–20). In addition, emerging evidence indicates that APOE-ε*4* may affect motor performance and neuromuscular function, contributing to measurable changes in muscle strength, motor coordination, and overall physical performance (21–24). These effects suggest a broader influence of APOE-ε*4* beyond cognitive decline and AD risk, highlighting its potential role in neuromuscular degeneration and motor system vulnerability.

Research has shown that individuals carrying at least one copy of the ε*4* allele experience a faster rate of motor decline in older age, with a two-fold increase compared to non-carriers (21). This accelerated decline is largely attributed to changes in muscle strength, suggesting that APOE-ε*4* may influence neuromuscular function through its effects on muscular performance (21). APOE-ε*4* effects on motor function can be linked to APOE-ε*4*-mediated mechanisms such as mitochondrial dysfunction, oxidative stress, and disrupted synaptic signaling, all of which can impair motor performance and contribute to accelerated motor decline (25).

While gross motor tasks primarily involve larger muscle groups and may overlook subtle deficits, speech production, which relies on highly coordinated, continuous activation of fine motor units, presents a unique opportunity to detect early motor impairments in APOE-ε*4* carriers, potentially preceding overt cognitive or physical symptoms. Converging evidence shows that measurable subclinical changes in speech-related muscle activities can be detected early in neurodegeneration (26, 27). Speech production is a complex behavior that depends on the coordinated interaction of central and peripheral neural systems, respiratory support, phonation, articulation, and neuromuscular control. Consequently, characterization of speech-related dysfunction in neurological disease increasingly relies on multidimensional assessment frameworks that integrate complementary physiological and behavioral measures rather than a single marker alone. In disorders such as amyotrophic lateral sclerosis (ALS), multidisciplinary motor speech protocols incorporate perceptual, acoustic, respiratory, electrophysiological, and functional assessments to capture the heterogeneous manifestations of bulbar impairment and improve disease characterization (28). Such frameworks recognize that different assessment modalities provide distinct yet complementary information regarding underlying neural and neuromuscular mechanisms.

Although speech has emerged as a promising source of digital biomarkers in AD (29–34), most studies have focused primarily on acoustic, linguistic, or cognitive measures, with comparatively limited attention to the neuromuscular processes underlying speech production. Surface electromyography (EMG) offers a direct window into these processes by quantifying muscle activation patterns and informing motor unit behavior during speech. The present study therefore investigates the influence of the APOE-ε*4* allele on neuromuscular function in speech-related craniofacial muscles using surface EMG, aiming to contribute a neuromuscular dimension to the emerging multidimensional framework for speech-based assessment of APOE-ε*4*-related vulnerability.

The primary objective was to evaluate whether surface EMG measures extracted from craniofacial muscles during speech could differentiate APOE-ε*4* carriers from noncarriers in cognitively intact individuals. To further investigate potential mechanisms linking APOE-ε*4* to neuromuscular dysfunction, associations between EMG measures and blood-based lipid and metabolic markers were examined. These biomarkers were selected to probe pathways related to mitochondrial function, oxidative stress, and metabolic activity that may contribute to altered orofacial neuromuscular function in APOE-ε*4* carriers. Given the well-documented effects of APOE-ε*4* on mitochondrial pathways, including oxidative stress, impaired ATP synthesis, and motor decline (21, 25, 35), characterizing the relationship between APOE-ε*4* pathology and neuromuscular dysfunction may provide insight into early physiological alterations associated with genetic vulnerability to AD. Standard neuropsychological measures were included to characterize the cognitive status of the cohort and to provide clinical context, rather than to serve as a directly comparable classification benchmark for the EMG-based models.

It was hypothesized that carrying the APOE-ε*4* allele may contributes to motor unit remodeling or loss, resulting in altered neuromuscular efficiency. These alterations could plausibly manifest as atypical motor unit recruitment and firing patterns during speech tasks, potentially reflected by increased complexity, greater variability, or the adoption of compensatory strategies. Such neuromuscular changes may be characterized by modified EMG signal amplitudes and frequency shifts, indicating altered conduction velocities and disrupted motor unit synchronization. Furthermore, the APOE-ε*4* allele may influence central motor pathways, affecting the functional connectivity between neural structures such as brain-to-muscle interactions or coordination between functionally related muscle groups. These effects can be assessed using coherence measures obtained from EMG-EEG or EMG-EMG signal analyses. A reduction in coherence may suggest impaired neural signal integration during motor tasks. By investigating EMG patterns in speech-related muscles, this study aims to elucidate the impact of APOE-ε*4* on neuromuscular function, generating preliminary evidence regarding EMG-based markers of APOE-ε*4*-related vulnerability that may ultimately contribute to hypothesis generation about neuromuscular features that could, in future work, be evaluated as potential biomarkers in relation to early diagnosis, disease monitoring, and personalized therapeutic interventions.

## 2 Methods

The study received approval from the MGB Institutional Review Board (IRB Protocol #2021P001460), and all participants provided written informed consent to participate.

### 2.1 Participant population

Forty-two adults participated in this study. Participants were divided into two groups based on their APOE genotype: APOE*-*ε*4* positive (ε4+); (*n* = 16) and APOE*-*ε*4* negative (ε4-) (*n* = 26). The ε4+ group included individuals with ε3/ε4 heterozygous (*n* = 13) and ε4/ε4 homozygous (*n* = 3) genotypes, while the ε4- group consisted of individuals with the ε3/ε3 homozygous genotype. The groups were matched for age, and education (>12 years). APOE genotyping was conducted using blood samples, and all participants were blind to their respective genotypes. Of the 42 participants, 28 (7/5 female/male in the ε4+ group; 4/12 female/male in the ε4- group) had available blood-based lipid, metabolic, hematological, and vitamin/mineral markers, from which a subset was selected based on their relevance to mitochondrial function, metabolism, and oxidative stress. These data, along with demographic and clinical information for both groups are summarized in Table 1.

**Table 1:** Demographic and clinical characteristics stratified by genotype carrier status.

Variables	All participants	ε4^−^	ε4^+^	
	(*n* = 42)	(*n* = 26)	(*n* = 16)	*p*-value
Female, *n* (%)	17 (40.48)	8 (30.77)	9 (56.25)	0.102^**†**^
Family history of AD, *n* (%)	21 (50.00)	13 (50.00)	8 (50.00)	1.00^†^
Age, mean (*SD*)	68.26 (7.16)	69.73 (7.67)	65.88 (5.69)	0.070^‡^
Number of years of education, mean (*SD*)	16.24 (2.09)	16.54 (1.88)	15.75 (2.38)	0.270^‡^
MMSE, mean (*SD*)	29.17 (1.08)	29.19 (1.17)	29.12 (0.96)	0.840^‡^
MoCA, mean (SD)	27.29 (2.27)	27.46 (2.21)	27.00 (2.39)	0.537^‡^
Speech intelligibility, mean (SD)	99.01 (1.10)	98.84 (1.06)	99.34 (1.16)	0.247^‡^
Speaking rate, mean (SD)	185.16 (23.33)	182.12 (19.13)	190.24 (29.06)	0.346^‡^
	**All participants**	ε**4**^−^	ε**4**^+^	
**Blood-based biomarkers**	**(*****n*** = **25)**	**(*****n*** = **15)**	**(*****n*** = **10)**	***p*** **-value**
Blood Glucose Level, mean (SD)	92.44 (14.69)	91.40 (17.71)	94.00 (9.12)	0.635
HbA1c, mean (SD)	5.43 (0.38)	5.41 (0.37)	5.45 (0.43)	0.827
Hemoglobin, mean (SD)	13.84 (1.03)	14.11 (0.90)	13.44 (1.13)	0.132
Triglycerides, mean (SD)	101.12 (63.97)	112.80 (77.07)	83.60 (33.22)	0.209
Total Cholesterol, mean (SD)	184.20 (38.42)	180.13 (35.91)	190.30 (43.14)	0.546
Total Cholesterol/HDL Ratio, mean (SD)	3.12 (0.64)	3.13 (0.70)	3.09 (0.57)	0.866
LDL-Cholesterol, mean (SD)	104.12 (32.99)	100.40 (32.97)	109.70 (33.96)	0.506
Folate, Serum, mean (SD)	16.51 (5.31)	17.13 (4.91)	15.29 (6.25)	0.511
Vitamin B12, mean (SD)	488.21 (265.54)	447.71 (294.85)	544.90 (220.22)	0.365
Calcium, mean (SD)	9.30 (0.37)	9.43 (0.26)	9.10 (0.44)	0.050

*SD*, standard deviation; MMSE, mini-mental state examination; MoCA, montreal cognitive assessment; HbA1C, hyperglycemia A1C.

^†^Chi-square test.

^‡^*t*-test.

All participants met the following inclusion criteria: (1) capacity to provide informed consent and comply with task-specific study instructions; (2) native speakers of American English; (3) no previous record of speech, language, hearing, psychiatric, or neurological disorders; (4) no clinical diagnosis of MCI; (5) not currently using psychoactive drugs that could potentially impact study outcomes; (6) passed an age-adjusted pure-tone hearing screening, ensuring normal hearing acuity in at least one ear; and (7) dental and occlusion status within functional limits, as determined by an oral examination performed by ME. Additionally, all participants demonstrated normal functional speech as assessed through the Sentence Intelligibility Test (SIT), a standardized tool that measures speech intelligibility and speaking rate. During the assessment, participants read 11 sentences randomly generated by the SIT software, with sentence length progressively increasing from 5 to 15 words. Their speech was digitally recorded and subsequently transcribed orthographically by two native American English-speaking listeners with established high interrater reliability (ICC = 0.99, *p* < 0.001). The SIT software calculated speech intelligibility as the percentage of correctly transcribed words and speaking rate as the number of words read per min (WPM). Furthermore, participants exhibited no sound distortions or abnormalities in voice, resonance, prosody, or rate qualities, as independently judged by two expert raters (ME and PR).

### 2.2 Experimental tasks

Participants were presented with nine sentences and instructed to read each sentence a fixed number of times. They produced five repetitions of the sentence “Buy Bobby a puppy” and two repetitions of each of the remaining eight sentences. These sentences were: “Billy blows big blue bubbles”; “Busy buzzing bumble bees”; “Bake big batches of bitter brown bread”; “Bankers pull plenty of bills into banks”; “Betty picked plenty of apple bits from the baby's bib”; “Blue balloons pop when placed above sharp spikes”; “Ben's brother, Patrick, beat up Ben's pal, Bill”; and “Paul baked plenty of baguettes but put butter on the bottom.”

The sentences were selected because they include speech sounds that engage seven targeted speech muscles, including two lip muscles (inferior and superior orbicularis oris of the lip) and five jaw muscles (left and right masseter, left and right temporalis, and anterior belly of the digastric (ABD)), all of which are realiably accessible by surface electrodes (36–38). Specifically, the sentences are particularly rich in bilabial consonants (/b/, /p/, /m/), which require substantial activation of the orbicularis oris muscles. For instance, “Buy Bobby a Puppy” and “Billy blows big blue bubbles” prominently feature these sounds, making them ideal for observing the coordinated action of facial muscles. Similarly, the plosives (/b/, /p/) and fricatives (/f/, /v/) in phrases “Bake big batches” and “Blue balloons pop” engage the orbicularis oris, ABD and other facial muscles in rapid and precise movements. Alveolar sounds (/t/, /d/, /n/) in words like “Betty,” “plenty,” and “bottom” involve significant activation of the temporalis and masseter muscles for jaw positioning and stabilization. These sounds also recruit the ABD muscles for jaw opening, particularly in transitions between consonants and vowels. Phrases like “plenty” and “spikes” include consonant clusters that demand complex coordination across articulatory muscles, offering further insights into dynamic muscle control. The phonetic structure of sentences such as “Busy buzzing bumble bees” promotes repeated activation of the targeted craniofacial muscle groups. Additionally, the sentences feature a diverse range of vowel sounds, requiring varied configurations of the oral cavity and corresponding activation patterns of the speech muscles, including the ABD for jaw movement during low and mid vowels (39). While phonetically rich, these sentences also closely resemble natural speech, eliciting more realistic muscle activation patterns than those produced during isolated phoneme tasks. In summary, the combination of diverse consonant types and vowel contexts generates a comprehensive range of muscle activation patterns, making these stimuli particularly effective for EMG analysis of speech production.

### 2.3 Neuropsychological assessment

Participants underwent a comprehensive array of standard neuropsychological tests routinely used to characterize AD-related conditions which assess attention, processing speed, learning, memory, language, and executive function. Cognitive tests used in this study included montreal cognitive assessment (MoCA), Mini-Mental Status Examination (MMSE), trail making tests A and B, the Hopkins Verbal Learning Test (HVLT), and the Controlled Oral Word Association Test (COWAT).

The *MoCA* is a widely used cognitive screening tool designed to evaluate a broad range of cognitive abilities and is particularly sensitive to detecting MCI and early-stage dementia (40, 41). It provides a comprehensive assessment across multiple cognitive domains, including attention and concentration, executive functioning, memory, language, visuospatial abilities, orientation, and abstract thinking. Attention and concentration are assessed through digit spans, vigilance, and serial subtraction, while executive functioning is evaluated using tasks such as trail-making exercises and abstraction questions. Memory is measured through short-term recall of a word list, reflecting encoding and retrieval processes. Language is assessed through naming, sentence repetition, and verbal fluency tasks, while visuospatial abilities are tested using clock drawing and cube copying exercises. Orientation is evaluated through questions about the date, time, and place, and abstract thinking is measured by identifying similarities between items. The MoCA provides a total score out of 30, with lower scores indicating potential cognitive impairment. A score below the cutoff (typically 26) suggests the need for further assessment or intervention.

The *MMSE* is a widely used cognitive screening tool designed to assess global cognitive function and detect potential cognitive impairment (42, 43). This brief, standardized test evaluates various cognitive domains, providing an overall measure of cognitive abilities. The MMSE assesses orientation by asking individuals to identify the date, day of the week, and their current location, reflecting awareness of time and place. Registration is tested by asking participants to repeat a list of three unrelated words, while attention and calculation are evaluated through tasks such as serial sevens (subtracting 7 repeatedly from 100) or spelling a word backward, measuring concentration and working memory. Delayed recall of the three words presented earlier assesses memory. Language abilities are tested through tasks like naming common objects, repeating sentences, following instructions, and writing a sentence, evaluating expressive and receptive language skills. Visuospatial skills are assessed by asking participants to copy a simple geometric figure, measuring visual perception and motor coordination. The MMSE provides a total score out of 30 points, with lower scores indicating greater cognitive impairment. A score of 24 or below is often used as a cutoff to indicate cognitive deficits, though adjustments may be made based on age, education, and cultural factors.

The *TMT*, comprising two parts (A and B), is a widely used neuropsychological assessment designed to evaluate various cognitive abilities, including visual attention, task switching, processing speed, and executive functioning (44, 45). In Part A, participants connect a sequence of randomly arranged numbers in ascending order by drawing a line as quickly and accurately as possible. This segment primarily measures visual scanning, numerical sequencing, and motor speed. Part B requires participants to alternate between different cognitive tasks or mental sets, assessing cognitive flexibility, divided attention, and the inhibition of impulsive responses, while also incorporating the skills evaluated in Part A (46, 47). Longer completion times and increased errors on the TMT are indicative of cognitive impairments and can affect everyday activities, including driving. Although the TMT is often scored using a ratio of Part B to Part A or by adjusting Part B scores relative to Part A, this analysis focused on evaluating each part independently. This approach allows for a more comprehensive exploration of distinct cognitive demands, encompassing both higher-order and lower-order processes.

The *HVLT* is a widely used measure of verbal learning and memory, consisting of multiple subtests that assess various aspects of cognitive functioning (48, 49). These include immediate recall, delayed recall, and retention efficiency, which provide insights into encoding, storage, and retrieval processes in memory. The HVLT Immediate Total Recall subtest evaluates a participant's ability to immediately recall a list of words presented verbally over multiple learning trials. It reflects short-term memory capacity, attention, and the initial encoding of verbal information. Performance on this measure is influenced by the ability to organize and retain information during the learning process. The HVLT Delayed Recall component assesses the participant's ability to recall the same word list after a delay, without re-presentation of the list. It provides information on the strength of memory retention over time and the ability to retrieve information from long-term memory. Lower performance can indicate challenges with memory storage or retrieval. Finally, the HVLT Percent Retention measures the proportion of words recalled during the delayed recall phase relative to the words initially learned (immediate recall). It serves as an indicator of memory decay and retention efficiency. Higher retention percentages suggest more effective memory consolidation, while lower percentages may highlight difficulties in retaining learned information over time. These HVLT subtests, when analyzed together, offer a comprehensive understanding of verbal memory performance, enabling the identification of patterns associated with encoding, retention, and retrieval deficits.

The *COWAT*, specifically the FAS variant, is a widely used measure of phonemic verbal fluency designed to assess language production and executive functioning (50). This test examines the participant's ability to generate words under specific phonological constraints, providing insights into cognitive processes such as mental flexibility, retrieval strategies, and inhibitory control. In this task, participants are asked to produce as many words as possible within a given time frame (typically 60 s per letter) that begin with a specified letter (e.g., F, A, or S) excluding proper nouns (i.e., name of people of places), numbers, and derivatives of the same word (i.e., repetitions of the same word with different affixes). Performance on this test evaluates the ability to organize and retrieve words based on phonemic rules while suppressing irrelevant or automatic responses. The FAS test relies on multiple cognitive domains, including language skills (retrieval of vocabulary and phonological processing), executive functioning (strategic planning, mental flexibility, and switching between subcategories within the letter constraint), and inhibitory control (suppressing inappropriate responses that do not meet the criteria). Reduced word generation or clustering, such as producing words only within a narrow subcategory, may indicate impairments in cognitive flexibility, processing speed, or access to lexical stores. Performance on the FAS test is commonly used in neuropsychological evaluations to identify deficits associated with frontal lobe dysfunction, neurological conditions, or disorders that impact executive functioning and language production.

All tests were scored using standardized norms adjusted for demographic variables including age, sex, education level, and ethnicity, when appropriate, to calculate z-scores for each test. Subsequently, the cognitive composite z-score for each participant was calculated by summing the six standardized performance scores (i.e., TMT A, TMT B, HVLT Immediate Total Recall, HVLT Delayed Recall, HVLT Percent Retention, COWAT-FAS).

To evaluate clinical status, participants' cognitive profiles were assessed using a set of established neuropsychological diagnostic frameworks, with the actuarial Jak/Bondi criteria serving as the pre-specified primary decision rule. This approach requires converging evidence of impairment across multiple tests within a domain, rather than classifying MCI based on a single impaired score, and has been shown in multiple cohorts to yield more stable diagnostic classifications, and stronger associations with AD biomarkers and progression than conventional Petersen/Winblad single-test criteria (51, 52). We additionally applied several commonly used, more liberal frameworks (Conservative Criteria and Subtype, Custom Criteria and Subtype, and conventional Petersen Criteria and Subtype) to describe the distribution of single-domain amnestic and non-amnestic profiles across APOE-ε*4* carriers and noncarriers. These supplementary frameworks were not used to define the primary cognitive status of the cohort and were instead treated as secondary sensitivity descriptions, because conventional Petersen/Winblad-style criteria rely on a single impaired score within a domain and are susceptible to false-positive MCI classifications, whereas actuarial Jak/Bondi criteria require at least two impaired scores within a domain and generally improve diagnostic specificity and stability (51, 53, 54).

All neuropsychological data were administered, scored, and interpreted by an experienced neuropsychologist. Administration of neuropsychological batteries and EMG recordings took place either on the same day (for 12 participants) or on different days (for 30 participants), depending on the participant's availability and preference. For sessions conducted on separate days, the interval between cognitive and EMG assessments averaged approximately 3 months (range: 2 weeks to 6 months), thereby maintaining reasonable temporal proximity between cognitive and EMG data.

### 2.4 EMG recording

EMG data collection was conducted in the Speech physiology and neurobiology of aging and dementia (SPaN-AD) lab at MGH Institute of Health Professions. Participants were seated in a chair, with speech tasks presented in a quasi-random order on a screen approximately 6 feet away. Each participant was fitted with surface electrodes for myoelectric data acquisition and a head-mounted microphone for acoustic data collection as described below.

EMG data were collected bilaterally from multiple facial and jaw muscles using a wireless EMG system (BIOPAC). Self-adhesive bipolar Ag/AgCl electrodes (15 mm diameter, 20 mm inter-electrode distance) were placed on the upper lip (two electrodes), lower lip (two electrodes), anterior belly of digastric (ABD, two electrodes), masseter (four electrodes, two pairs on each side), and temporalis (four electrodes, two pairs on each side). A ground electrode was attached behind the ear over the mastoid process.

Prior to electrode placement, the skin was prepared using an alcohol swab to increase conductance. Electrodes were positioned using craniofacial anatomical landmarks for reproducibility: along the cantho-gonial line for masseter, vertically at the coronal suture for anterior temporalis, and submentally along the posterior-inferior direction for ABD. Electrode placement was verified through calibration tasks such as jaw oscillations and clenching to ensure proper capture of muscle activities.

The EMG signals were pre-amplified by 1,000, band-pass filtered at 10 Hz−500 Hz, digitized at 10,000 Hz using the MP150 data acquisition module, and recorded using AcqKnowledge software (BIOPAC Systems, Inc.). Acoustic data used to annotate the EMG recordings were acquired using a Williams Sound MIC094 head-mounted microphone placed approximately 5 cm from the left lip corner. The audio signal was processed through a Focusrite Scarlett 2i2 audio interface and digitized at 10,000 Hz, synchronously with the EMG data. Participants were instructed to read sentences at their habitual speaking rate and loudness. The simultaneous recording of myoelectric and acoustic data allowed for temporal alignment during subsequent analysis.

### 2.5 EMG data processing and analysis

All EMG data processing and analytic procedures were implemented in MATLAB (55) using custom-developed scripts. Following the best-practice guidelines for EMG signal processing (56), all seven channels were notch-filtered at 60 Hz and high-pass filtered at 20 Hz to remove power line noise and low-frequency artifacts, respectively. Lastly, the EMG signals for all repetitions of each stimulus were concatenated, resulting in 375 data samples (42 participants × 9 stimuli − 3 errors = 375) in total.

The processed EMG data samples were analyzed using an integrative automated analytic approach that adopted and combined two recently developed and validated EMG analytic algorithms, originally designed for a different neurodegenerative population (26, 37). This method extracts 70 features across the seven EMG channels. An overview of these features is outlined in Table 2. This analytic approach combined linear and nonlinear analyses in time and frequency domains to measure the frequency, amplitude, and complexity characteristics of individual muscle activities, as well as the functional connectivity of the whole craniofacial muscle network. These features have previously been shown to capture disease-related neuromuscular changes in motor unit recruitment, firing, and synchronization in craniofacial musculature in ALS (1, 2) and were therefore adopted here to assess hypothesized APOE-ε*4*-associated alterations in craniofacial neuromuscular performance.

**Table 2:** Overview of surface electromyography features.

Feature	Feature notation	# Features	Assessed construct
Frequency-domain analysis			
Median frequency	MDF_RMAS, MDF_LMAS, MDF_RTEMP, MDF_LTEMP, MDF_UL, MDF_LL, MDF_ABD	7	Frequency
Coherence	str_RMAS, str_RMAS, str_LMAS, str_RTEMP, str_LTEMP, str_UL, str_LL, str_ABD	7	Functional connectivity
Time-domain analysis			
Mean absolute value	MAV_RMAS, MAV_LMAS, MAV_RTEMP, MAV_LTEMP, MAV_UL, MAV_LL, MAV_ABD	7	Amplitude (overall)
Mean absolute value slope	MAVSlp_RMAS, MAVSlp_LMAS, MAVSlp_RTEMP, MAVSlp_LTEMP, MAVSlp_UL, MAVSlp_LL, MAVSlp_ABD	7	Amplitude (dynamics)
Waveform length	WL_RMAS, WL_LMAS, WL_RTEMP, WL_LTEMP, WL_UL, WL_LL, WL_ABD	7	Amplitude (complexity)
Zero crossing rate	ZR_RMAS, ZR_LMAS, ZR_RTEMP, ZR_LTEMP, ZR_UL, ZR_LL, ZR_ABD	7	Frequency
Slope sign change	SlpSignChange_RMAS, SlpSignChange_LMAS, SlpSignChange_RTEMP, SlpSignChange_LTEMP, SlpSignChange_UL, SlpSignChange_LL, SlpSignChange_ABD	7	Frequency
Time-frequency domain analysis			
Shannon entropy	ShanEn_RMAS, ShanEn_LMAS, ShanEn_RTEMP, ShanEn_LTEMP, ShanEn_UL, ShanEn_LL, ShanEn_ABD	7	Regularity
Recurrence quantification analysis			
Recurrence rate	RR_RMAS, RR_LMAS, RR_RTEMP, RR_LTEMP, RR_UL, RR_LL, RR_ABD	7	Complexity
Determinism	DET_RMAS, DET_LMAS, DET_RTEMP, DET_LTEMP, DET_UL, DET_LL, DET_ABD	7	Complexity

MDF, median power spectral frequency; str, strength of functional connectivity; MAV; mean absolute value; MAVSlp, slope of mean absoluate value; WL, waveform length; ZR, zero crossing rate; SlpSignChange, slope sign change; ShanEn, Shannon entropy; RR, recurrence rate; DET, determinism; RMAS, right masseter; LMAS, left masseter; RTEMP, right temporalis; LTEMP, left temporalis; UL, upper lip muscles; LL, lower lip muscles; ABD, anterior belly of diagstric.

#### 2.5.1 Frequency-domain analysis

The frequency-domain analysis calculated the power spectral density of each EMG signal and the coherence spectra between different EMG signals. Power spectral density was estimated by Welch's method, which was implemented by 1,024-point Fast Fourier Transform (FFT) applied over a sliding 100-msec, 50% overlap Hamming window (57). The median frequency (MDF) of power spectral density was calculated as the frequency that divided the power spectrum into two parts with equal integrated spectral energy.

To calculate coherence spectra, all EMG signals were full wave rectified and reconstructed by concatenating stationary 1-s epochs around the bursts for each channel. Magnitude-squared coherence spectrum was calculated for each pair of reconstructed signals,, using 8,192-point FFT applied over a sliding 8,192-point Hamming window with 75% overlap, based on the Equation 1:


$$\begin{array}{l} |R_{xy}{|}^{2}=\frac{{{|S}_{xy}\left(f\right)|}^{2}}{S_{xx}{\left(f\right)S}_{yy}\left(f\right)} \end{array}$$ (1)

where ${\left|R_{xy}\right|}^{2}$ is magnitude-squared coherence spectrum, *S*_*xy*_(*f*) is the cross-spectrum between two muscles, and *S*_*xx*_(*f*) and *S*_*yy*_(*f*) are the auto-spectra for each muscle. In addition, a significance level corresponding to the upper 95% confidence limit under the hypothesis of independence between muscles was calculated as: $S=1-0.05^{1/\left(\hat{L}-1\right)}$, where $\hat{L}$ is the adjusted number of overlapped segments in coherence calculation (58, 59). The significance level was used to exclude spurious functional connections between muscles.

The present study focused on coherence within the beta band. Beta oscillations (15 Hz−35 Hz) have been suggested to originate from the motor cortical network, providing a descending drive for synchronous modulation of functionally related muscles (60). Thus, beta-band coherence can be interpreted as a measure of functional connectivity between muscles related to a common cortical drive. To measure beta-band coherence, the significance of the coherence spectrum within the beta band was first verified relative to the significance level derive above. Following signficance verification, the mean coherence values within the beta band were computed and transformed to Fisher z-scores for variance stabilization (58). Lastly, the mean strength of functional connectivity was calculated for each muscle as the average sum of Fisher z-score of beta-band coherence between this and all other muscles.

#### 2.5.2 Time-domain analysis

The time-domain analysis was applied to 100-msec segments centered on activation bursts, where bursts were defined as intervals during which the level of activation remained above a participant- and muscle-specific reference value, represented by the mean activation level across the recording. The features extracted were then averaged across all segments for each channel. Such short segments represent the initial phase of muscle contraction, which encodes the most stable and useful neuromuscular information (61). This analysis extracted five features for each muscle, including

(1) Mean absolute value (MAV), defined as the mean absolute value of all data points within a segment, as shown in Equation 2:


$$\begin{array}{l} MAV=\frac{1}{N}\sum_{i=1}^{N}|x_{i}| \end{array}$$ (2)

where N is the number of data points in the segment and *x*_*i*_ is ith data point in the segment.

(3) Mean absolute value slope (MAVSlp), defined as the difference of MAV between adjacent segments, as shown in Equation 3:


$$\begin{array}{l} \begin{array}{c} MAVSlp_{j}=MAV_{j+1}-MAV_{j} \end{array} \end{array}$$ (3)

where *j* = 1, 2, …*J*−1 and *J* is the total number of segments in the EMG signal.

(3) Zero crossings (ZR), defined as the number of times the waveform crosses zero, as shown in Equation 4:


$$\begin{array}{l} ZR=\overset{N-1}{\underset{i=1}{\sum }}\left[f\left({-x}_{i}\times x_{i+1}\right)\times |x_{i}-x_{i+1}|\geq 4\;mV\right] \end{array}$$ (4)

where $f\left(x\right)=\{\begin{array}{c} 1,x>0\\ 0,x\leq 0 \end{array}$ . To eliminate noise fluctuations, a 4 *mV* threshold (i.e., 4 μ*V* peak-to-peak intrinsic system noise × 2, 000 gain/2) is implemented while calculating ZR.

(4) Slope sign change (SlpSignChange), defined as the number of times the slope of the waveform changes sign, as shown in Equation 5:


$$\begin{array}{l} SlpSignChange=\overset{N-1}{\underset{i=2}{\sum }}f\left[\left(x_{i}-x_{i-1}\right)\times \left(x_{i}-x_{i+1}\right)\geq 4\;mV\right] \end{array}$$ (5)

Like for ZR, a 4 *mV* threshold is employed to reduce noise-induced slope sign changes.

(5) Waveform length (WL), defined as the cumulative length of the waveform over the segment, as shown in Equation 6:


$$\begin{array}{l} WL=\overset{N-1}{\underset{i=1}{\sum }}|x_{i+1}-x_{i}| \end{array}$$ (6)

#### 2.5.3 Time-frequency domain analysis

First, each EMG signal was decomposed into eight scale-frequency representations, using 3-level wavelet packet decomposition with the 5th order Symlets wavelet (62). Next, the Shannon entropy of each time-frequency representation was computed using Equation 7:


$$\begin{array}{l} ShanEn=-\sum_{i}{s_{i}}^{2}\log\left({s_{i}}^{2}\right) \end{array}$$ (7)

where *s*_*i*_ is the ith coefficient of the scale-frequency representation, reflecting the approximation (i.e., low-pass) or detail (i.e., high-pass) coefficients at each decomposition level. Lastly, the sum of the Shannon entropy of all eight scale-frequency representations was calculated to index the irregularity of myoelectric activity for each muscle.

#### 2.5.4 Recurrence quantification analysis (RQA)

RQA quantified the structural complexity of EMG signals based on the dynamics of state change in the recurrence plot (63). A recurrence plot is composed of recurrence points, where a recurrence point is defined as a state *j* when its phase space trajectory ${\vec{x}}_{j}$ returns to a previous state i so that the distance between ${\vec{x}}_{j}$ and ${\vec{x}}_{i}$ is below the threshold ε. Here the phase space trajectory ${\vec{x}}_{j}$ is defined as: ${\vec{x}}_{j}=\left[u_{j},u_{j+\tau },\ldots u_{j+\left(m-1\right)\tau }\right]$, where *m* is the embedding dimension parameter, and τ is the time delay parameter. Recurrent plots were generated for non-overlapping 1-s segments of each EMG signal, with the parameters set to the following pre-determined values: *m* = 30, τ = 3, ε = 0.2. Such segments represented stationary intervals of EMG signals, allowing for reliable analysis of the underlying motor unit firing patterns. Two quantitative metrics, recurrence rate (RR) and determinism (DET), were derived from each segment and then averaged across all segments for each muscle. RR measured the ratio of all recurrence points to all possible states in the recurrence plot. DET measured the ratio of recurrence points forming diagonal structures to all recurrence points, which can be interpreted as an index of the overall periodic content in the signal.

### 2.6 Statistical analysis

All statistical analyses were conducted in the R statistical computing program (64), with the significance level set to α < 0.05. Given the exploratory nature of this study and the modest sample size, inferential results were treated as hypothesis-generating.

#### 2.6.1 Effect of APOE-ε4 on neuromuscular function

To provide an initial inspection of the effect of APOE*-*ε*4* on neuromuscular function, the effect sizes of the difference between all EMG features for APOE*-*ε*4* carriers and noncarriers were calculated using Cohen's d. Due to the differential impact of APOE*-*ε*4* on males and females (65–67), the effect sizes were calculated for the two sexes separately.

#### 2.6.2 Factor analysis

To reduce the dimension of the EMG feature set, domain-specific exploratory factor analysis (*fa*) with *oblimin* rotation was applied to sentence-level observations (*N* = 375) to cluster features that shared common variance into a lower-dimensional set of latent factors, where the number of factors was determined by parallel analysis (*fa.parallel*) (68). This procedure aimed to circumvent the curse of dimensionality, as commonly encountered in digital health applications (69), to avoid overfitting to a specific dataset in the subsequent classification analysis. Given the potential dependency among within-participant observations related to task repetition, a participant-level factor analysis was conducted in parallel using the within-participant median of each feature to verify the feature loading patterns and factor structure derived from the sentence-level analysis.

Features were divided into five domains: (1) functional connectivity between muscles, (2) right-sided jaw muscles, (3) left sided jaw muscles, (4) submental muscles, and (5) lip muscles. This division was based on both theoretical knowledge that suggests different innervation patterns of jaw and lip muscles and empirical evidence showing partially independent functioning of right and left-sideded craniofacial muscles (ref). Factor analysis was conducted within each domain to cluster relevant features into factors. These factors represented unobserved neuromuscular traits of the craniofacial muscle network. Factor scores were estimated using the *tenBerge* method, providing EMG-based measures of participants' neuromuscular performance in the latent factor space.

#### 2.6.3 Predictive power of EMG-based measures for classifying APOE-ε4 carriers from noncarriers

To evaluate the differences in EMG-based measures between APOE*-*ε*4* carriers and noncarriers, each EMG-based measure was submitted to a linear mixed effects model, with genotype (i.e., binary variable denoting APOE*-*ε*4* carriers vs. noncarriers), sex, age, and the interaction between genotype and sex as the fixed effects, and a participant-dependent intercept term as the random effect. The main effect of APOE*-*ε*4* and its interaction with sex were the primary effects of interest for evaluating the significance of carrier-noncarrier difference.

In parallel, to evaluate the differences in standard measures derived from the cognitive domain (i.e., scores on MoCA, MMSE, and the comprehensive z-score of all domain-specific neuropsychological tests) between APOE*-*ε*4* carriers and noncarriers, all cognitive measures underwent a similar analysis as above. Specifically, general linear models were used to evaluate the effects of genotype, sex, age, and the interaction between genotype and sex on each cognitive measure. These cognitive analyses were used to characterize the cognitive status of the cohort and explore potential genotype-related differences, rather than to construct directly comparable classification models to the EMG-based framework.

In addition, the discriminatory efficacy of the combined EMG-based measures for differentiating APOE-ε*4* carriers from noncarriers was evaluated. To this end, all EMG-based measures, along with age and sex, were fed into shrinkage discriminant analysis (SDA), a regularizerd variant of the broader discriminant analysis family for reducing overfitting risk (70), to classify between sentence-level observations for carriers and noncarriers. To obtain robust estimates of model performance, repeated nested participant-level cross-validation was employed. The outer loop consisted of 10 repetitions of stratified 5-fold cross-validation, in which participants, rather than individual observations, were randomly partitioned into five mutually exclusive folds while preserving the class distribution. All observations from a given participant were assigned to the same fold to prevent information leakage between training and testing sets. Within each outer training set, hyperparameter optimization was performed using an inner participant-level leave-one-participant-out cross-validation procedure. The hyperparameters included a numeric variable (lambda) specifying the degree of shrinkage and a logical parameter specifying the covariance structure (i.e., full or diagonal). Model tuning was conducted using the *train* function in the *caret* package, with a tuning grid of 10 candidate hyperparameter combinations, and the optimal model was selected based on cross-validated classification accuracy. Prior to model fitting, all predictors were centered and scaled using parameters estimated exclusively from the training data within each resampling iteration.

After hyperparameter optimization, the optimal SDA model was refitted to the entire outer training set and evaluated on the corresponding held-out outer test participants. Classification performance was evaluated using accuracy, sensitivity, specificity, positive predictive value (PPV), and negative predictive value (NPV), reported as the mean and 95% confidence interval calculated from the cross-validated results. In addition, the receiver operating characteristic (ROC) curve, along with its area under the curve (AUC), was computed for visual examination of classification performance. Given the relatively small sample size and the internal validation design, these estimates are interpreted as exploratory and hypothesis-generating rather than as definitive diagnostic accuracy.

#### 2.6.4 Correlation of EMG-based measures with blood-based biomarkers

Spearman's correlation coefficients were calculated between all EMG-based measures and blood-based biomarkers. The magnitudes of the correlation coefficients were interpreted to evaluate the relation between the biolological and neuromuscular factors, in order to identify potential biological underpinnings of the APOE*-*ε*4*-induced neuromuscular changes.

## 3 Results

### 3.1 Cognitive classification of the cohort

Cognitive status was summarized using the pre-specified actuarial Jak/Bondi criteria as the primary framework. As shown in Table 3, all participants (16 APOE-ε4 carriers and 26 noncarriers) were classified as cognitively normal under Jak/Bondi criteria, and no individuals met MCI criteria. The Jak/Bondi subtype framework likewise identified no impaired subtypes; all 42 participants were classified as normal.

**Table 3:** Cognitive classifications across diagnostic frameworks by APOE-ε4 carrier status.

Framework	Classification	APOE-ε4 carriers (*n* = 16)	Noncarriers (*n* = 26)	Total (*n* = 42)
Bondi criteria	Normal	16	26	42
	MCI	0	0	0
Bondi subtype	Normal	16	26	42
	MCI subtype	0	0	0
Conservative criteria	Normal	14	24	24
	MCI	2	2	2
Conservative subtype	Normal	14	24	24
	MCI subtype	2 (Single-Amnestic)	2 (Single-Amnestic)	2
Custom criteria	Normal	15	26	41
	MCI	1	0	1
Custom subtype	Normal	15	26	41
	MCI subtype	1 (Single-Non amnestic)	0	1
Petersen criteria	Normal	14	20	34
	MCI	2	6 (Single-Amnestic)	8
Petersen subtype	Normal	14	20	34
	MCI subtype	2 (Single-Amnestic)	6 (Single-Amnestic)	8

For descriptive purposes, we also applied more liberal alternative frameworks to summarize cognitive status across the cohort. Under the Conservative Criteria, 24 participants were classified as normal (14 carriers, 10 noncarriers) and 4 were classified as MCI (2 carriers and 2 noncarriers), all with single-domain amnestic profiles. Under the Custom Criteria, 41 participants were classified as normal (15 carriers, 26 noncarriers) and 1 was classified as MCI (1 carrier), with a single-domain non-amnestic profile. Under the Petersen Criteria, 34 participants were classified as normal (14 carriers, 20 noncarriers) and 8 were classified as MCI (2 carriers, 6 noncarriers), all with single-domain amnestic profiles. The corresponding subtype frameworks (Conservative, Custom, and Petersen Subtype) reflected these same single-domain amnestic or non-amnestic patterns among individuals meeting criteria within each framework.

Overall, all participants were cognitively normal under the primary actuarial Jak/Bondi framework, whereas a small subset met MCI criteria only under the more liberal exploratory frameworks. The distribution of these alternative classifications was broadly similar across APOE-ε4 carriers and noncarriers.

### 3.2 Effect of APOE-ε4 on EMG features

The effect size of the difference between the EMG features for APOE-ε*4* carriers and noncarriers is displayed by sex in Figure 1. Exploratory sex-stratified Cohen's d values suggested both common and distinct patterns in neuromuscular measures for males and females.

**Figure 1:**
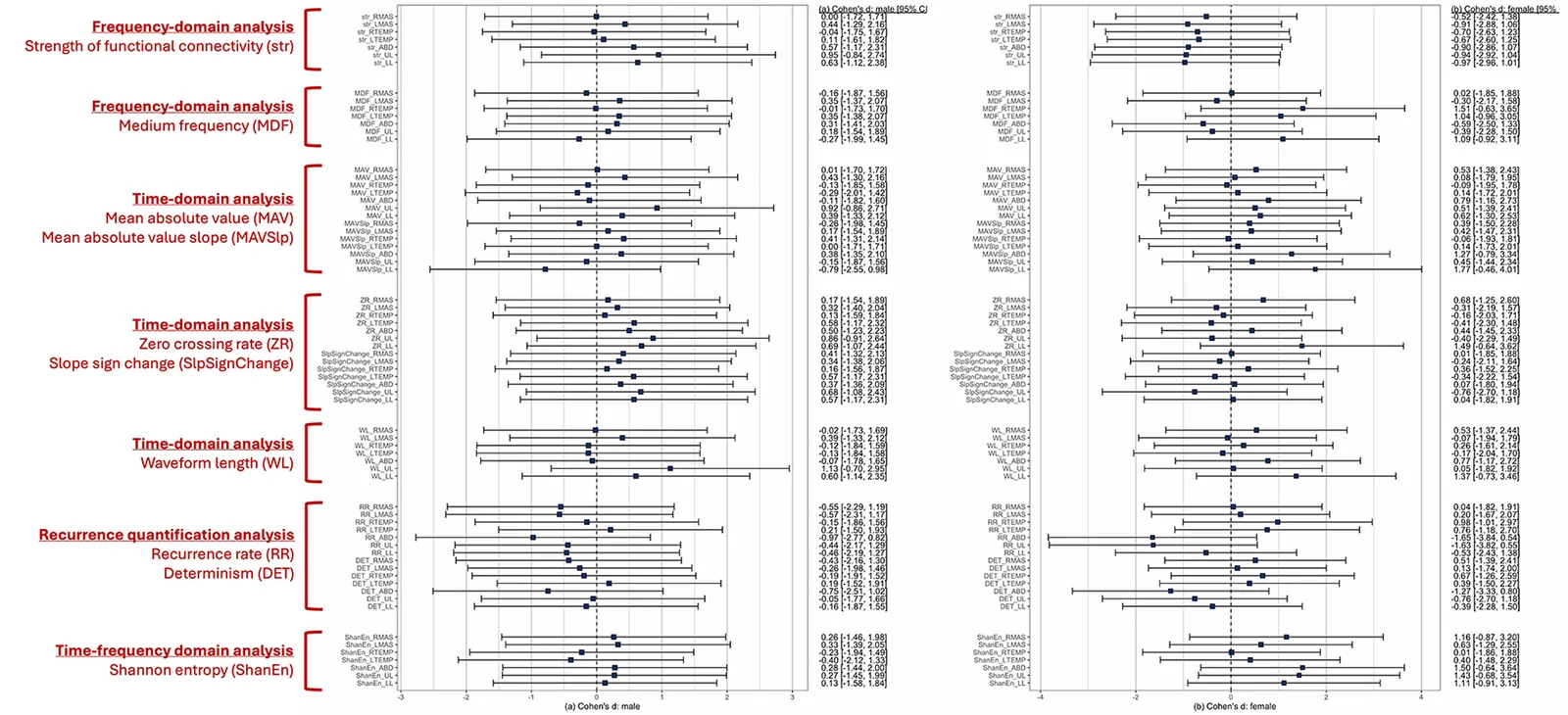
Cohen's d effect size of the difference between the surface electromyograph features for **(a)** male APOE-ε*4* carriers and noncarriers; **(b)** female APOE-ε*4* carriers and noncarriers. RMAS, right masseter, LMAS, left masseter, RTEMP, right temporalis, LTEMP, left temporalis, ABD, anterior belly of digastric, UL, upper lip muscles, LL, lower lip muscles.

Focusing on the features that exhibited at least a medium effect size (|d| > 0.50), the common effects of APOE-ε*4* for both sexes included (1) increased amplitude (i.e., increased MAV and WL), especially for lip muscles, (2) increased frequency (i.e., increased MDF, ZR, SlpSignChange), and (3) increased complexity (i.e., decreased RR and DET and increased ShanEn). The distinct effects of APOE-ε*4* for males and females were primarily manifested in functional connectivity. Specifically, male APOE-ε*4* carriers exhibited a trend toward increased functional connectivity (i.e., increased str), whereas female APOE-ε*4* carriers showed an opposite trend toward reduced functional connectivity (i.e., decreased str), relative to their noncarrier counterparts.

### 3.3 Factor analysis

Using sentence-level factor analysis, the 70 EMG features across the five domains were clustered into 14 latent factors based on the scree plots generated by parallel analysis, as shown in Figure 2. The rotated factor loadings are shown in Figure 3. The feature loading patterns and factor structure remained stable in the participant-level analysis (see Supplementary Figure S1), providing evidence for the validity of the sentence-level analysis.

**Figure 2:**
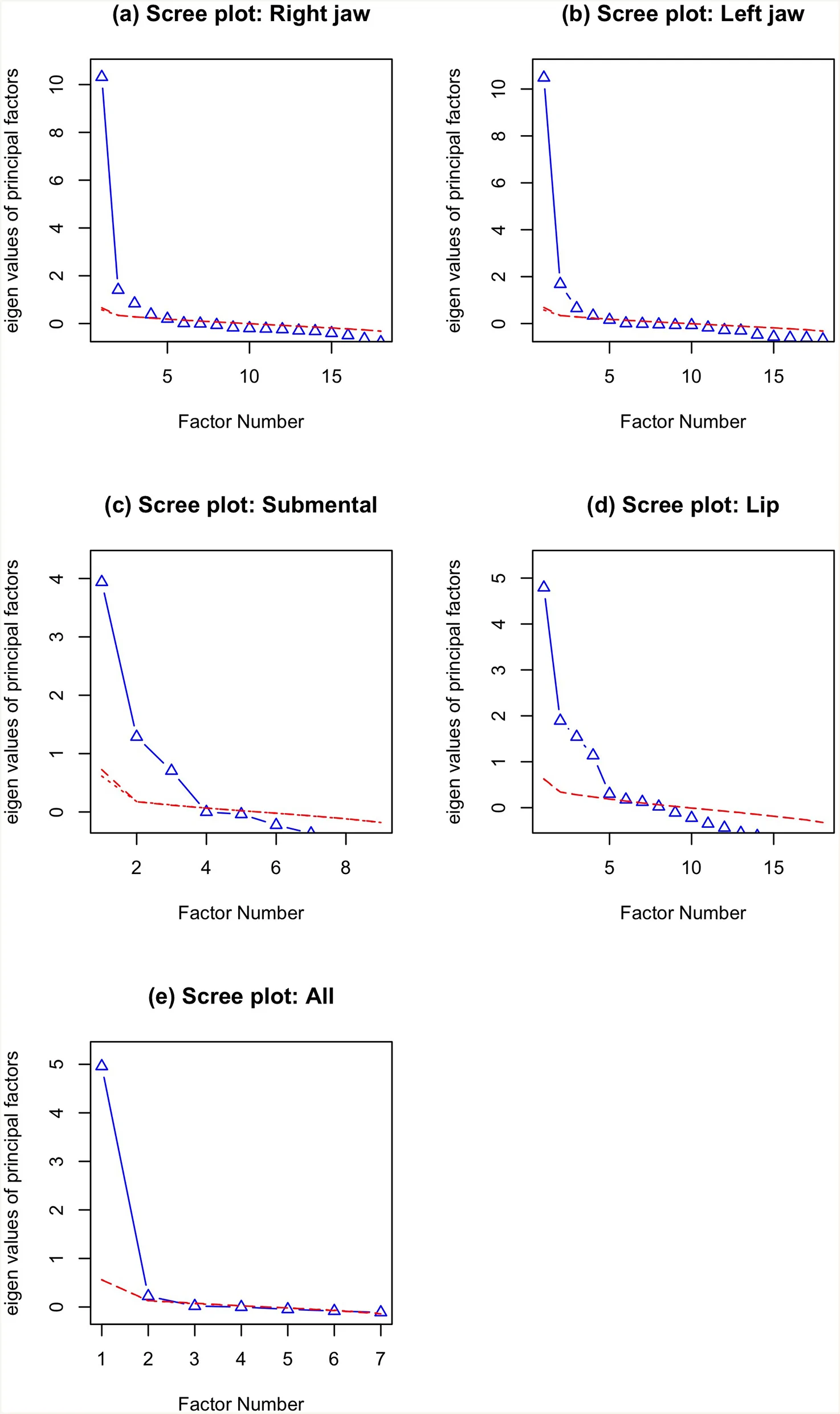
Scree plots generated by parallel analyses for five EMG feature categories: **(a)** right-sided jaw muscles, **(b)** left-sided jaw muscles, **(c)** submental muscles, **(d)** lip muscles, and **(e)** functional connectivity between all muscles. Blue triangles: actural data; red dashed line: simulated data.

**Figure 3:**
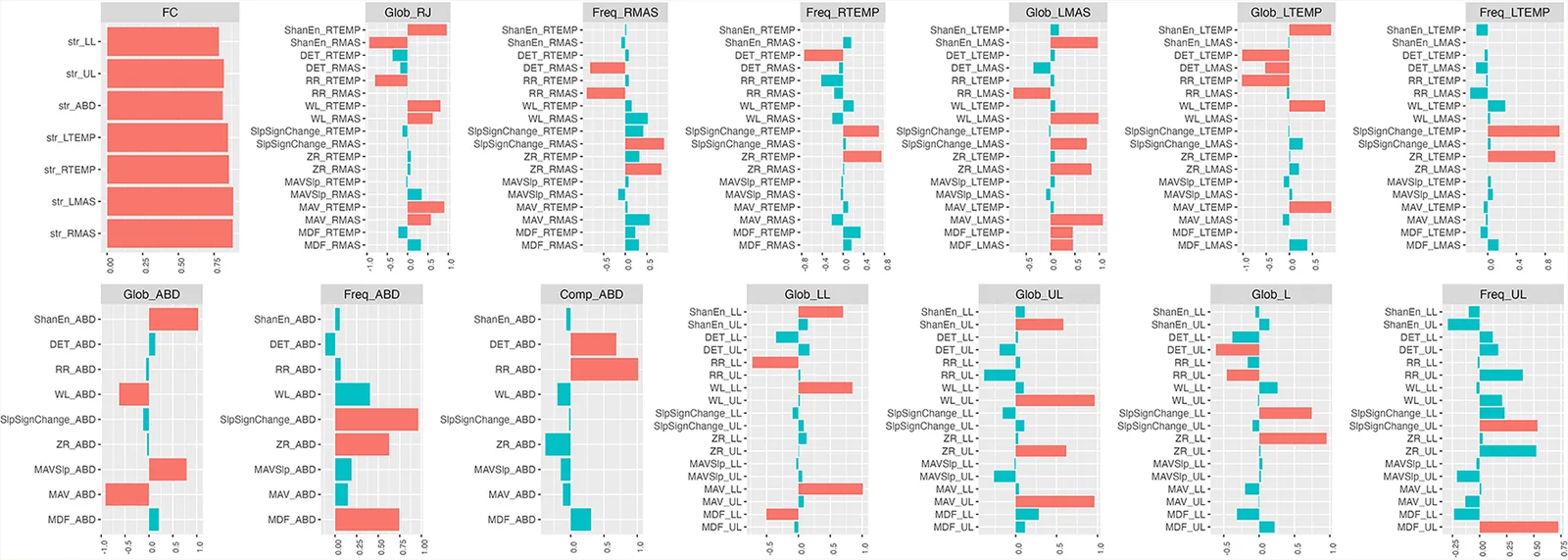
Rotated factor loadings derived from sentence-level factor analysis. The height of the bars represents the loading of the surface electromyography features on each latent factor. By convention, features that load greater than 0.4 (i.e., absolute value >0.4) are regarded as the primary component features of the latent factor, and the loadings of these features are shown in red, while the loadings of the other features are shown in blue.

As shown in Figure 2, features that loaded greater than 0.4 (i.e., absolute factor loading > 0.4) were conventionally regarded as the primary component features of the factor. Based on the feature loading patterns, all 14 latent factors were interpretable. These factors included (1) one reflecting the overall functional connectivity of the craniofacial muscle network (FC); (2) three reflecting right-sided jaw muscle characteristics (Glob_RJ: global characteristics of right masseter and right temporalis; Freq_RMAS: frequency of right masseter; Freq_RTEMP: frequency of right temporalis); (3) three reflecting left-sided jaw muscle characteristics (Glob_LMAS: global characteristics of left masseter; Glob_LTEMP: global characteristics of left temporalis; Freq_LTEMP: frequency of left temporalis); (4) three reflecting submental muscle characteristics (Glob_ABD: global characteristics of ABD; Freq_ABD: frequency of ABD; Comp_ABD: complexity of ABD); and (5) four reflecting lip muscle characteristics (Glob_LL: global characteristics of lower lip muscles; Glob_UL: global characteristics of upper lip muscles; Glob_L: global characteristics of upper and lower lip muscle characteristics; Freq_UL: frequency of upper lip muscles).

### 3.4 Discriminatory power of EMG-based measures for classifying APOE-*ε4* carriers from noncarriers

Of all 14 EMG-based measures, genotype exhibited a significant effect on the measure reflecting the complexity of ABD (Comp_ABD), *F*(1, 37.00) = 6.92, *p* = 0.012, and the measure reflecting the global characteristics of lip muscles (Glob_L), *F*(1, 37.01) = 5.35, *p* = 0.026; sex showed a significant effect on the measure reflecting the frequency of ABD (Freq_ABD), *F*(1, 37.00) = 6.79, *p* = 0.013; age revealed a significant effect on the measure reflecting the global characteristics of right-sided jaw muscles (Glob_RJ), *F*(1, 37.00) = 4.13, *p* = 0.049; whereas the interaction between genotype and sex showed a significant effect on the measure reflecting the frequency of upper lip muscles, *F*(1, 37.00) = 5.55, *p* = 0.024. Figure 4 shows the box plots for all EMG-based measures by genotype and sex. Of the three standard cognitive measures, no significant effect was found for genotype, sex, or the interaction between genotype and sex, whereas age showed a significant effect on the z-score of MMSE, *F*(1, 37) = 10.81, *p* < 0.001.

**Figure 4:**
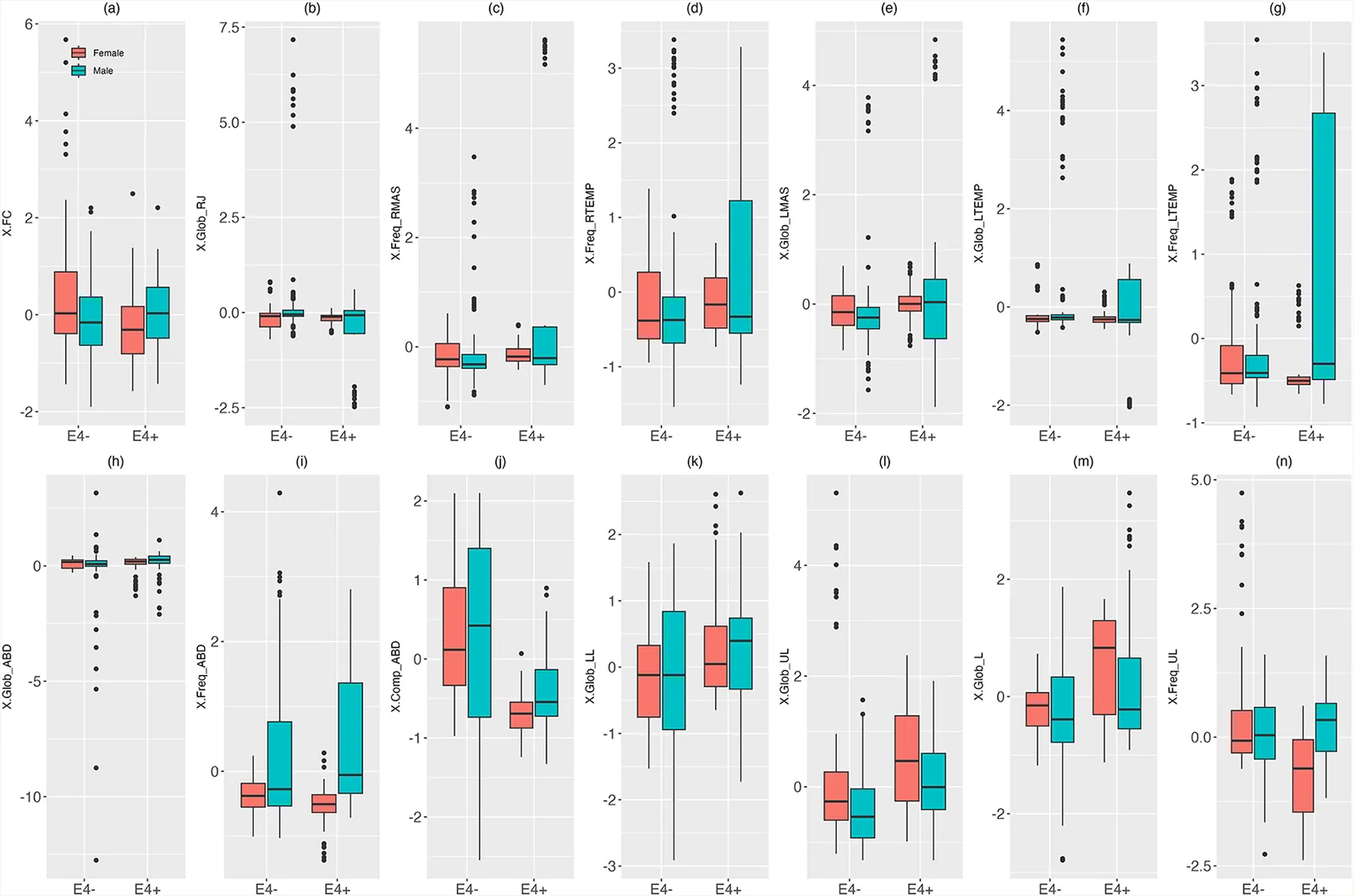
Box plots for EMG-based measures by genotype (E4+: APOE-ε4 carriers; E4_−_: APOE-ε4 noncarriers) and biological sex: **(a)** functional connectivity, **(b)** global characteristics of right masseter and right temporalis, **(c)** frequency of right masseter, **(d)** frequency of right temporalis, **(e)** global characteristics of left masseter, **(f)** global characteristics of left temporalis, **(g)** frequency of left temporalis, **(h)** global characteristics of anterior belly of digastric, **(i)** frequency of anterior belly of digastric, **(j)** complexity of anterior belly of digastric, **(k)** global characteristics of lower lip muscles, **(l)** global characteristics of upper lip muscles, **(m)** global characteristics of upper and lower lip muscles, and **(n)** frequency of upper lip muscles.

The ROC curve for the multivariate classification model based on all EMG-based measures is shown in Figure 5. The model achieved a mean AUC of 0.73, accuracy of 0.64, sensitivity of 0.71, specificity of 0.52, PPV of 0.72, and NPV of 0.57. The median of the final hyperparameter lamda across all folds and repetitions was 0.44, with a full covariance structure. The out-of-fold confusion matrix is reported in Table 4, and the mean and confidence intervals for the classification performance metrics are reported in Table 5.

**Figure 5:**
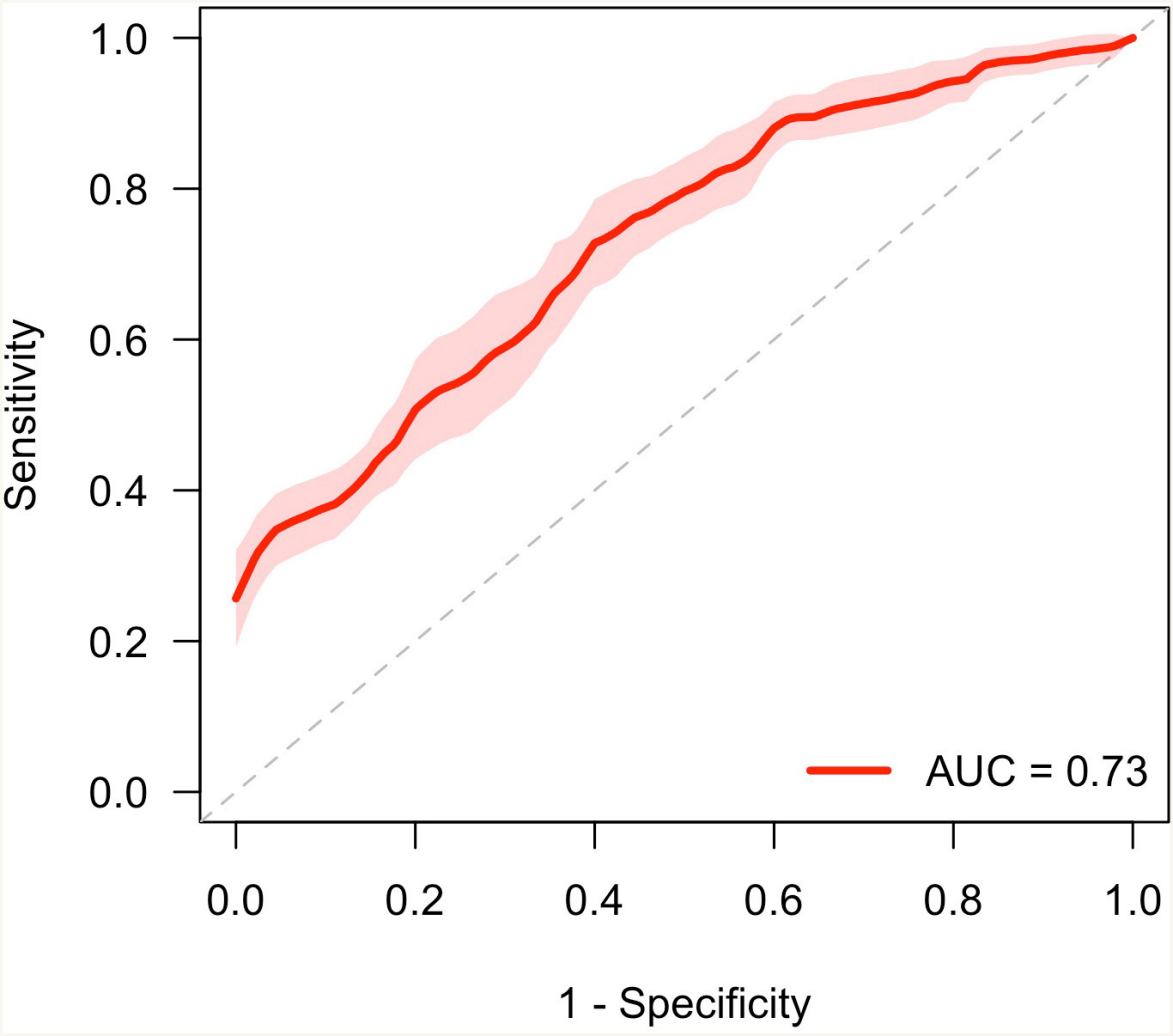
Receiver operating characteristic (ROC) curve for the classification between APOE-ε*4* carriers and noncarriers, generated from repeated nested participant-level cross-validation of shrinkage discriminant analysis. The solid line and its surrounding shaded region correspond to the mean ROC curve and the 95% confidence interval, respectively. AUC, area under the curve.

**Table 4:** Out-of-fold confusion matrix for multivariate classification based on repeated nested participant-level cross-validation.

	ε4-	ε4+
**ε4-**	1,660	687
**ε4+**	670	733

Row: prediction; column: reference. E4_−_: APOE-E4 noncarriers, E4_+_: APOE-E4 carriers

**Table 5:** Performance metrics for multivariate classification based on repeated nested participant-level cross-validation.

	Mean	95% CI
Accuracy	0.64	[0.61, 0.66]
Sensitivity	0.71	[0.68, 0.75]
Specificity	0.52	[0.46, 0.58]
PPV	0.72	[0.69, 0.75]
NPV	0.57	[0.53, 0.60]

CI, confidence interval; PPV, positive predictive valie; NPV, negative predictive value.

### 3.5 Correlation of EMG-based measures with blood-based biomarkers

As shown in Figure 6, multiple blood-based biomarkers, including the total and LDL cholesterol, hemoglobin, and vitamin B12, were correlated with selective EMG-based measures. Using an uncorrected significance level of *p* < 0.05, the amplitude and complexity of lower lip muscles (captured by Glob_LL) were associated with the total and LDL cholesterol. An increase of Glob_LL (reflecting increased amplitude and complexity of lower lip muscles, as observed in APOE-ε*4* carriers from Figures 1, 3) was correlated with decreased total and LDL cholesterol. The frequency and complexity of lip muscles (captured by Glob_L) were associated with vitamin B12, with an increase of Glob_L (reflecting increased frequency of lower lip muscles and increased complexity of upper lip muscles) correlated with increased vitamin B12. ABD characteristics (captured by Glob_ABD and Freq_ABD) were associated with hemoglobin, with increased Glob_ABD (reflecting primarily increased complexity) and decreased Freq_ABD (reflecting reduced frequency) correlated with reduced hemoglobin. The global characteristics of left masseter (captured by Glob_LMAS) were associated with hemoglobin, with an increase of Glob_LMAS (reflecting increased amplitude, complexity, and frequency) correlated with reduced hemoglobin. The frequency of left temporalis (captured by Freq_LTEMP) was also associated with hemoglobin, with an increase of Freq_LTEMP (reflecting increased frequency) correlated with increased hemoglobin. The frequency of right temporalis (captured by Freq_RTEMP) was associated with hemoglobin and LDL cholesterol. An increase of Freq_RTEMP (reflecting increased frequency) was correlated with increases in both hemoglobin and LDL cholesterol. However, since none of these correlations survived the multiplicity correction using the false discovery rate method (corrected *p*≥0.63), the observed relationships between the EMG-based measures and blood-based biomarkers should be considered exploratory and be interpreted cautiously.

**Figure 6:**
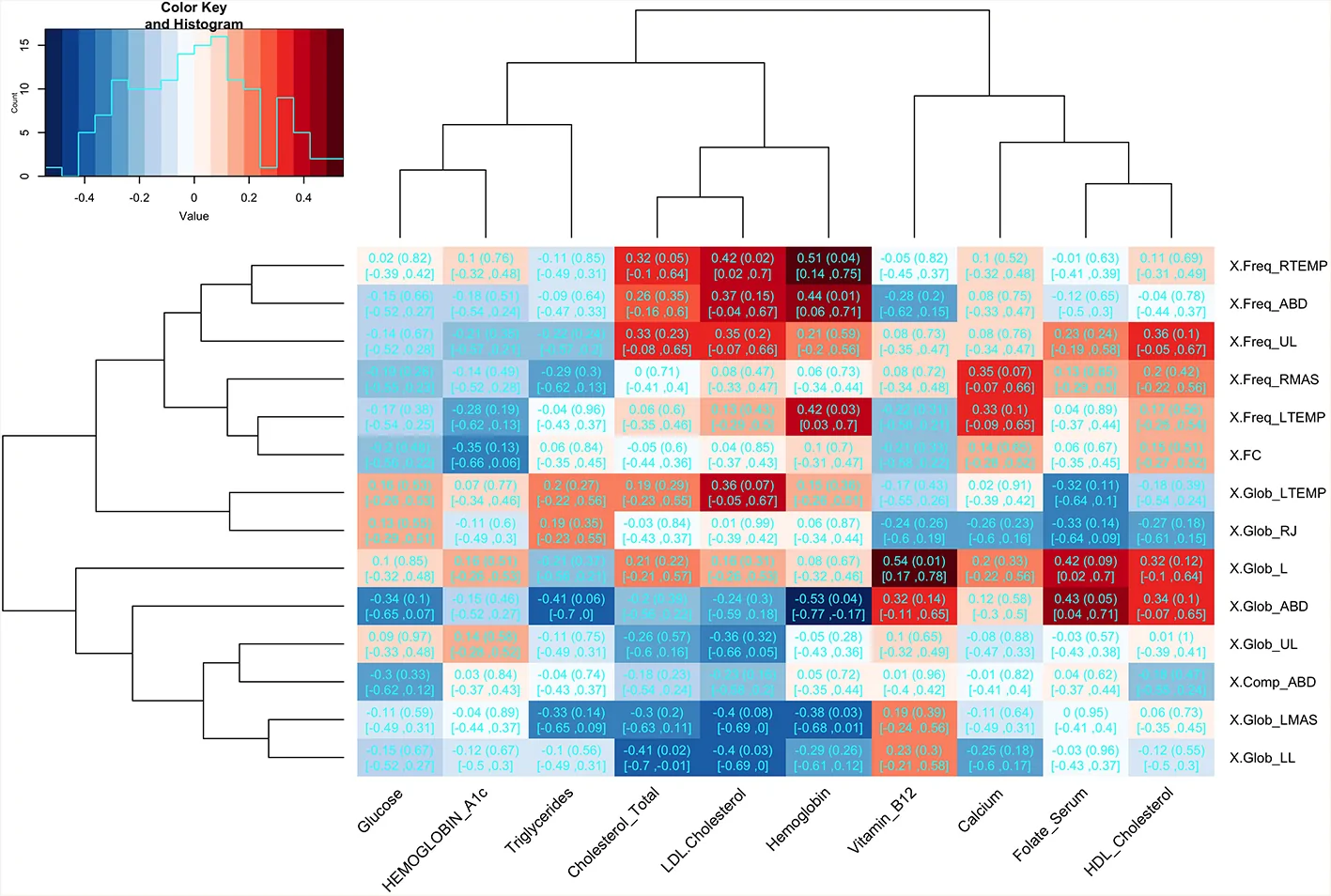
Heatmap for Spearman's correlation coefficients between the EMG-based measures (in rows) and blood-based biomarkers (in columns). The numeric values within each cell are the correlation coefficients, uncorrected *p*-values (in parentheses), and 95% confidence intervals (in brackets).

## 4 Discussion

This study investigated the influence of APOE*-*ε*4*, a known genetic risk factor for AD, on the neuromuscular function of speech-related muscles. Features extracted from craniofacial muscles showed promising, internally validated exploratory discrimination in classifying APOE*-*ε*4* carriers from noncarriers. Increased motor unit recruitment and synchronization emerged as candidate neuromuscular risk factors for AD. These alterations may reflect APOE-ε*4*-related changes in central and peripheral neuromuscular control, potentially linked to mitochondrial dysfunction, oxidative stress, and impaired cellular metabolism, but any increase in muscle fatigability should be considered a plausible hypothesis rather than a confirmed outcome, as fatigue was not directly measured in this study.

### 4.1 Speech-based neuromuscular risk factors for AD

Although APOE-ε*4* is a known risk factor for cognitive decline and AD, its effects on motor function remain underexplored. This study reveals altered neuromuscular function in APOE-ε*4* carriers during speech, marked by increased amplitude, frequency, and complexity of myoelectric signals across facial and jaw muscles (Figure 1). These patterns suggest a possible shift in motor unit recruitment strategies, characterized by increased activation of larger, fast-conducting motor units and greater synchronization of smaller motor units. Although such changes are often observed under conditions of neuromuscular fatigue (57, 71), they should be interpreted here as indicative of altered or compensatory neuromuscular control rather than direct evidence of fatigue, as fatigue was not explicitly measured in this study. Such compensatory recruitment may help sustain force production but could also contribute to premature fatigue and altered motor control dynamics. It may further lead to higher-frequency muscle activity (72) and increased heterogeneity in the physiological properties of the recruited motor unit pools, resulting in more complex firing patterns.

### 4.2 Biological correlates of neuromuscular changes

The proposed link between APOE-ε*4*-associated neuromuscular changes and increased fatigability is further supported by the observed biochemical correlates of EMG features; however, this relationship remains inferential and should be interpreted as hypothesis-generating rather than definitive, given the absence of direct fatigue measurements in this study. Key neuromuscular factors, including the global characteristics of the lip muscles, submental muscles, and the left masseter, as well as the frequencies of the bilateral temporalis muscles, correlate with total and LDL cholesterol, hemoglobin, and vitamin B12. These biomarkers have critical implications for mitochondrial function and oxidative stress.

Cholesterol shapes mitochondrial membrane composition, thereby influencing energy production and oxidative stress (73), processes often dysregulated in APOE-ε*4* carriers due to altered lipid metabolism (74). The accumulation of these metabolites can lead to increased group III and IV afferent feedback (75), potentially necessitating compensatory increases in motor unit recruitment and discharge rates to maintain task demands. Vitamin B12 plays essential roles in one-carbon metabolism and homocysteine regulation, affecting DNA synthesis, methylation reactions, and overall cellular energy balance (76, 77). Deficiencies in these vitamins can lead to elevated homocysteine levels, a known risk factor for cognitive decline, and may also contribute to reduced neuromuscular efficiency and greater susceptibility to muscle fatigability in APOE-ε*4* carriers (73). Finally, hemoglobin is essential for oxygen transport and mitochondrial ATP production (78). Impaired oxygen delivery can shift metabolism toward anaerobic glycolysis, promoting lactate accumulation and driving adaptations in motor unit behavior consistent with greater fatigability (73).

### 4.3 Cellular mechanisms underlying neuromuscular dysfunction in APOE-*ε4* carriers

At the molecular level, APOE-ε*4* disrupts mitochondrial pathways involved in electron transport, ATP synthesis, and thermogenesis (79), thereby impairing cellular energy homeostasis and potentially contributing to neuromuscular dysfunction and motor decline. Moreover, APOE-ε*4* carriers exhibit heightened oxidative stress, particularly in synaptosomal membranes, leading to neuronal damage, motor neuron degeneration, and astrocyte dysfunction (80, 81). The interplay of oxidative stress, mitochondrial inefficiency, and disrupted synaptic signaling might have collectively influenced neuromuscular function in APOE-ε*4* carriers in the current study, aligning with previous research (21, 82, 83).

Separately, the APOE-ε*4* allele significantly impacts calcium ion dynamics and excitation-contraction coupling, potentially disrupting motor unit recruitment and synchronization. Studies have linked APOE-ε*4* to mitochondrial calcium overload, demonstrated in Neuro-2A cells and rat hippocampal neurons (84, 85). This dysregulation compromises mitochondrial bioenergetics and ATP production, exacerbating oxidative stress and triggering pro-apoptotic pathways, contributing to neuronal and motor dysfunction (84).

Additionally, calcium homeostasis disruptions extend to the neuromuscular junction, where precise calcium signaling is essential for effective excitation-contraction coupling. APOE-ε*4*-associated calcium dysregulation may impair the timing and strength of muscle contractions, further exacerbating motor unit recruitment and synchronization deficits. Blood calcium levels were marginally lower in APOE-ε*4* carriers compared to noncarriers (*p* = 0.05). While blood calcium levels provide limited insight into intracellular calcium ion dynamics, this finding suggests a potential systemic effect of APOE-ε*4* on calcium homeostasis, warranting further investigation.

### 4.4 Neuronal hyperexcitability and neuromuscular compensation

Given the role of APOE-ε*4* in mitochondrial dysfunction, oxidative stress, and cellular metabolism, and the established links between these processes and increased fatigability, the altered muscle activity observed in APOE-ε*4* carriers may represent a compensatory response aimed at sustaining function in the context of emerging energy deficits and neuromuscular inefficiencies, rather than direct evidence of overt muscle fatigue. Supporting this, previous research indicates that iPSC-derived neurons from APOE-ε*4* carriers exhibit higher excitatory current frequencies compared to isogenic APOE-ε*3* control neurons (86). This hyperexcitability may further disrupt motor function by altering the balance between excitatory and inhibitory signaling within motor circuits. These metabolic and neuromuscular changes are consistent with a model in which APOE-ε4 increases vulnerability to muscle fatigue and drives adaptive shifts in motor unit behavior, but this mechanistic framework remains inferential given the cross-sectional design and lack of direct fatigue measures.

### 4.5 EMG-based neuromuscular markers as candidate indicators of AD vulnerability

APOE-ε*4*-driven changes in EMG measures, reflecting potential increases in motor unit recruitment and synchronization, may represent candidate neuromuscular correlates of AD vulnerability rather than established disease biomarkers. In this cognitively intact cohort, EMG-derived measures provided exploratory discriminatory information for APOE-ε4 carrier status, whereas standard cognitive tests did not show significant APOE-ε4–related group differences; however, these analyses were not designed to support direct, head-to-head comparisons of EMG-based vs. cognitive-based classification performance. Given the relatively small cross-sectional sample and internal validation design, any apparent differences between EMG and cognitive measures should be interpreted cautiously and as hypothesis-generating. Thus, further research in larger longitudinal cohorts is needed to determine whether EMG-derived measures can meaningfully complement existing neuropsychological approaches for risk stratification.

### 4.6 Sex-dependent impact of APOE-*ε4* on neuromuscular function

A sex-specific pattern was observed descriptively in craniofacial muscle connectivity (Figure 1). While female carriers showed an exploratory trend toward a global decrease, male carriers showed an exploratory trend toward an increase, relative to noncarriers. These descriptive EMG patterns are consistent with broader evidence from prior work indicating that female APOE-ε4 carriers experience a greater overall burden of AD-related pathology and a higher risk of progression to cognitive impairment than male carriers. Female APOE-ε4 carriers face a substantially higher risk of developing AD (87), show accelerated cognitive and functional decline with a greater likelihood of MCI and AD progression (88, 89), and exhibit more severe biomarker and pathological changes, including higher cerebrospinal fluid total tau, more pronounced Aβ deposition and tau accumulation, reduced glucose metabolism in key memory-related regions, faster rates of brain atrophy, and greater disruptions in brain connectivity and metabolic function (72–76). The reduced craniofacial functional connectivity observed in female carriers in the present study may reflect decreased integrity or efficiency of neuromuscular control networks supporting speech-related musculature, although this interpretation is hypothesis-generating and not directly proven by the current data. In contrast, the increased functional connectivity observed in male carriers may reflect relatively preserved motor unit pools and a putative capacity to recruit compensatory synchronous activation across craniofacial muscles to maintain performance.

The interaction between sex and APOE-ε*4* is likely driven by a complex interplay of genetic, hormonal (90), vascular (91, 92), and metabolic (92) factors. Notably, female APOE-ε*4* carriers show greater mitochondrial dysfunction(92), which may drive motor unit loss or remodeling, weakening muscle coupling and reducing functional connectivity. In males, the impact of APOE-ε*4* appears less pronounced as motor unit function is likely better preserved, providing a sufficient pool of intact motor units to maintain effective muscle coordination. The increased functional connectivity observed in male carriers may represent a compensatory adaptation, promoting greater synchronous muscle activation to augment system “stiffness” and potentially helping to stabilize orofacial movements and mitigate fatigue-related reductions in motor performance. It should be emphasized, however, that genotype-by-sex interaction terms in the EMG mixed-effects models were not statistically significant, and these sex-specific mechanistic interpretations should therefore be regarded as hypothesis-generating and not as evidence of an established diagnostic or prognostic biomarker.

Sex-specific differences in functional connectivity may limit its utility in distinguishing APOE-ε*4* carriers from noncarriers. Opposing trends in males and females can cancel each other out when analyzed together, reducing the discriminatory power of this measure. These findings highlight the importance of stratifying analyses by sex to better capture genotype-specific neuromuscular risk factors and to understand how APOE-ε*4*-related pathology differentially impacts speech motor control in women and men.

### 4.7 Differential effects of APOE-*ε4* on craniofacial musculature

This study reveals that APOE*-*ε*4* has an overall greater impact on facial than on mandibular muscles. Facial muscles, particularly those controlling lip movement, contain a higher proportion of fatigue-resistant, slow-twitch fibers and exhibit greater degrees of freedom, whereas mandibular muscles are more robust, with a larger contribution from fast-twitch, force-generating fibers (93). Additionally, lower facial muscles primarily receive contralateral cortical input, in contrast to the bilateral innervation of mandibular muscles (94). These anatomical and neurophysiological differences may make the facial musculature more vulnerable to subtle APOE-ε*4*-related neuromuscular disruption, helping to explain the more pronounced effects observed in lip muscles.

It is also important to note that speech production requires only moderate muscle activation, raising the question of whether the observed effects reflect APOE-ε*4* pathology, task-specific demands, or their interaction. In this study, the speech stimuli heavily featured bilabial plosives, requiring strong and precisely coordinated activation of lip muscles, but also involving jaw opening and closing movements. This may place a greater relative demand on lip muscles, potentially increasing their susceptibility to fatigue and making them appear disproportionately affected in APOE-ε*4* carriers. Consequently, while the observed pattern may partly reflect a task-induced effect, it remains unclear whether the greater involvement of facial vs. mandibular muscles directly results from APOE-ε*4* pathology or is influenced by the motor demands of the task. Further studies utilizing varied speech and non-speech motor tasks are needed to disentangle APOE-ε*4*-related changes from task-driven effects.

### 4.8 Limitations and future directions

Future studies using a wider range of speech stimuli and a larger sample size, especially of APOE-ε*4* carriers, are needed to evaluate the generalizability of the observed effects of APOE-ε*4* on orofacial muscles in the current study. Given the large number of correlated EMG features relative to the relatively small sample size in this study, factor analysis was applied to the full dataset rather than separately with the training datesets of each cross-validation iteration for the classification analysis. This approach was selected because the primary objective of this study was to explore and characterize the underlying structure of the EMG features and derive factors with fixed feature loading patterns that allow for evaluation of generalizability in future confirmatory analyses. Although this approach introduced potential information leakage, we considered it appropriate given the exploratory nature of the study and the objective of deriving empirically informed, interpretable constructs. Accordingly, classification performance should be interpreted as an estimate of the utility of the empirically derived constructs within this sample rather than as an unbiased estimate of prospective performance in an independent population. The resulting findings are therefore hypothesis-generating and require confirmation of the factor structure and predictive performance in independent samples in future studies.

In particular, because the cohort in this study was cognitively intact and no longitudinal follow-up data were collected, whether the EMG abnormalities observed here precede, track, or predict subsequent cognitive decline remains unclear. Thus, the present results should therefore be regarded as hypothesis-generating and not as evidence of an established diagnostic or prognostic biomarker. In addition, Figure 4 shows considerable variability in EMG measures within each genotype group. While sex and age were accounted for, other confounding factors may also contribute. Identifying these sources of variability will be key in future research. Beyond APOE-ε*4* and AD risk, EMG-based assessment of craniofacial and speech-related musculature may have broader applicability for quantifying neuromuscular changes in aging and in diverse neurological populations, such as individuals with stroke, progressive neuromuscular weakness, or muscular dystrophies (26, 95–97). However, the current protocol requires trained personnel for electrode placement, signal quality control, and offline signal processing, which may limit its immediate scalability to busy clinical environments. Although EMG-based indices indicate that craniofacial motor-unit recruitment and synchronization may serve as sensitive markers of subclinical neuromuscular dysfunction, it should be noted that, due to the lack of a maximum volunrary contraction (MVC) test, amplitude-based EMG measures are susceptable to inter-participant variability in electrode placement, skin-electrode impedance, subcutaneous tissue, anatomy, and task performance. Thus, the relationship between these measures and motor unit recruitment patterns should be interepreted as exploratory. The ultimate translation of these measures into clinical biomarkers requires further work to standardize task protocols and instrumentation, normalize EMG signals, and validate their performance across independent cohorts and longitudinal designs, before this approach can be implemented as a practical assessment tool complementing, rather than replacing, existing neuropsychological and clinical assessments beyond APOE-ε*4* and AD.

## 5 Conclusion

In conclusion, this study provides preliminary evidence that APOE-ε*4* carrier status is associated with measurable alterations in orofacial neuromuscular function in cognitively intact adults, characterized by various changes in EMG measures that together reveal potential increases in motor unit recruitment and synchronization, particularly in lip musculature that plays a central role in speech production. These genotype-related differences, which were observed descriptively as a sex-dependent pattern of craniofacial muscle connectivity, suggest that APOE-ε*4* may influence neuromuscular control of speech even before overt cognitive impairment emerges. These findings highlight the potential of EMG-based assessment of craniofacial and speech-related muscles as a sensitive, noninvasive approach for detecting early physiological changes associated with APOE-ε*4* carrier status, in a context where standard cognitive measures did not show significant group differences, and they generate testable hypotheses about potential links to AD vulnerability to guide future studies along this line. However, these neuromuscular signatures should be considered preliminary and hypothesis-generating; longitudinal and multimodal studies with clinical endpoints are needed to determine their prognostic value and establish whether they can serve as reliable early biomarkers of AD risk and how they relate to, or complement, established cognitive assessments.

## Data Availability

The raw speech recordings generated during the current study contain personally identifiable information and cannot be shared publicly for privacy and ethical reasons. A de-identified dataset comprising the raw EMG data and extracted EMG features that support the findings of this study will be available from the corresponding author upon reasonable request.
