# Supplementary material for: Exploratory associations between APOE-ε4 carrier status and facial neuromuscular activity in cognitively intact adults: a cross-sectional surface electromyography study
Source: Front Neurol. 2026 Sep 23;17:1740391. doi: 10.3389/fneur.2026.1740391 (PMC13642023; doi:10.3389/fneur.2026.1740391)

Supplemental Materials

Figure S1. Rotated factor loadings derived from participants-level factor analysis. The height of the bars represents the loading of the surface electromyography features on each latent factor. By convention, features that load greater than 0.4 (i.e., absolute value > 0.4) are regarded as the primary component features of the latent factor, and the loadings of these features are shown in red, while the loadings of the other features are shown in blue.

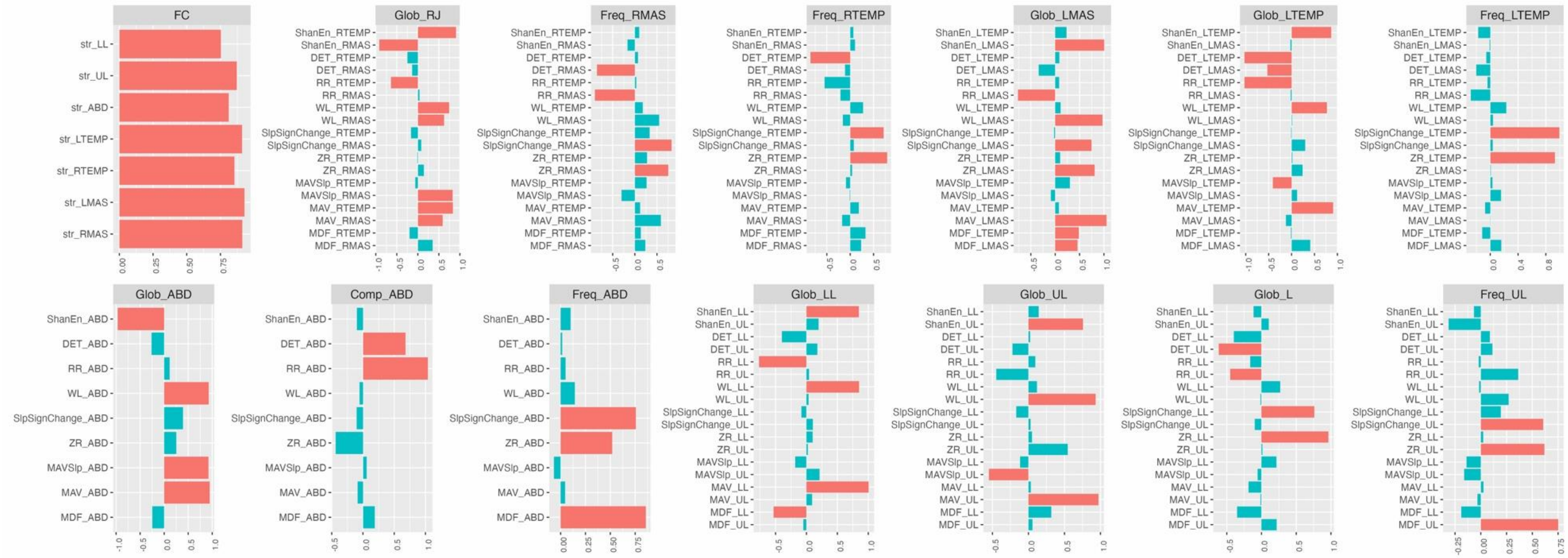

Supplement: Supplementary file 1 [file Data_Sheet_1.pdf]
